# The Phospholipase A1 Activity of Glycerol Ester Hydrolase (Geh) Is Responsible for Extracellular 2-12(*S*)-Methyltetradecanoyl-Lysophosphatidylglycerol Production in Staphylococcus aureus

**DOI:** 10.1128/msphere.00031-23

**Published:** 2023-03-28

**Authors:** Chitra Subramanian, Matthew W. Frank, My-Kyung Yun, Charles O. Rock

**Affiliations:** a Department of Infectious Diseases, St. Jude Children’s Research Hospital, Memphis, Tennessee, USA; The University of Iowa

**Keywords:** *Staphylococcus aureus*, phospholipid, glycerol ester hydrolase, phospholipase A1, membrane, virulence, fatty acids, phospholipids

## Abstract

Phosphatidylglycerol (PG) is the major membrane phospholipid of Staphylococcus aureus and predominately consists of molecular species with ≥16-carbon acyl chains in the 1-position and *anteiso* 12(*S*)-methyltetradecaonate (a15) esterified at the 2-position. The analysis of the growth media for PG-derived products shows S. aureus releases essentially pure 2-12(*S*)-methyltetradecanoyl-*sn*-glycero-3-phospho-1′-*sn*-glycerol (a15:0-LPG) derived from the hydrolysis of the 1-position of PG into the environment. The cellular lysophosphatidylglycerol (LPG) pool is dominated by a15-LPG but also consists of ≥16-LPG species arising from the removal of the 2-position. Mass tracing experiments confirmed a15-LPG was derived from isoleucine metabolism. A screen of candidate secreted lipase knockout strains pinpointed glycerol ester hydrolase (*geh*) as the gene required for generating extracellular a15-LPG, and complementation of a Δ*geh* strain with a Geh expression plasmid restored extracellular a15-LPG formation. Orlistat, a covalent inhibitor of Geh, also attenuated extracellular a15-LPG accumulation. Purified Geh hydrolyzed the 1-position acyl chain of PG and generated only a15-LPG from a S. aureus lipid mixture. The Geh product was 2-a15-LPG, which spontaneously isomerizes with time to a mixture of 1- and 2-a15-LPG. Docking PG in the Geh active site provides a structural rationale for the positional specificity of Geh. These data demonstrate a physiological role for Geh phospholipase A1 activity in S. aureus membrane phospholipid turnover.

**IMPORTANCE** Glycerol ester hydrolase, Geh, is an abundant secreted lipase whose expression is controlled by the accessory gene regulator (Agr) quorum-sensing signal transduction pathway. Geh is thought to have a role in virulence based on its ability to hydrolyze host lipids at the infection site to provide fatty acids for membrane biogenesis and substrates for oleate hydratase, and Geh inhibits immune cell activation by hydrolyzing lipoprotein glycerol esters. The discovery that Geh is the major contributor to the formation and release of a15-LPG reveals an unappreciated physiological role for Geh acting as a phospholipase A1 in the degradation of S. aureus membrane phosphatidylglycerol. The role(s) for extracellular a15-LPG in S. aureus biology remain to be elucidated.

## INTRODUCTION

Phospholipids are the major building blocks of biological membranes. Their deacylated forms are called lysophospholipids, which perform a number of signaling functions (1) and participate in deacylation-reacylation reactions that remodel the membrane phospholipid molecular species composition (2). Consistent with these functions, lysophospholipids are normally present in low abundance relative to bilayer forming phospholipids. Bacterial phospholipid acyl chain remodeling by a deacylation/reacylation cycle has not been described. Rather, the molecular species composition of bacterial membrane glycerolipids is governed by the acyl chain selectivity of the glycerol-phosphate acyltransferase pathways (either PlsB/C or PlsX/Y/C) (3). Bacterial lysophospholipids are known to arise from the use of phospholipids as acyl donors in biosynthetic pathways to other molecules (4). A well-studied example is the use of phosphatidylethanolamine (PE), the major membrane phospholipid of Escherichia coli, as a substrate for phospholipid:apolipoprotein transacylase (Lnt) (5, 6). Lnt catalyzes the transfer of the acyl chain at the 1-position of PE to the lipoprotein amino terminus. The use of PE as an acyl donor, rather than acyl-acyl carrier protein (ACP), is required because this terminal reaction in lipoprotein maturation occurs outside the cell. The resulting 2-acyl-*sn*-glycero-3-phosphoethanolamine (2-acyl-LPE) is transported into the cell interior by LplT (7) and the 1-position is acylated by the acyl-ACP dependent 2-acyl-LPE acyltransferase (8, 9). Staphylococcus aureus also uses membrane phospholipid as the substrate in the N-terminal acylation of lipoproteins by LnsAB, a heterodimeric phospholipid:apolipoprotein transacylase with the subunits derived from separate genes (10). S. aureus LnsAB would use phosphatidylglycerol (PG), the most abundant membrane phospholipid in this organism. The composition of lysophosphatidylglycerols (LPG) and how they are metabolized or reintroduced into the biosynthetic pathway has not been studied in S. aureus.

S. aureus is an important human pathogen that secretes a wide spectrum of protein factors that promote virulence (11, 12). Glycerol ester hydrolase (Geh, *SAUSA3300_0320*) is a well-known lipase that is one of the most abundant exoproteins (13) that is produced in nearly all S. aureus isolates (14). The transcription of the *geh* gene depends on the activity of the Agr quorum-sensing signal transduction system that acts as a master regulator of S. aureus virulence factor transcription (12). Geh has a role in virulence based on its hydrolysis of host lipids at the infection site to provide fatty acids for membrane biogenesis (15 to 18), substrates for oleate hydratase (19, 20), and by hydrolyzing lipoprotein glycerol esters to attenuate immune cell activation (21). The primary *geh* translation product is preproGeh, and the 37-residue signal sequence is removed concomitant with its transport out of the cell (22). The extracellular Geh (proGeh; 75 kDa) is cleaved by the aureolysin metalloprotease to mature Geh (mGeh; 44 kDa) that contains the catalytic triad (22, 23). The function of the pro-peptide is unclear because both forms appear equally active *in vitro* (23, 24) and both proGeh and mGeh are abundant exoproteins (23, 25). Because Geh is a secreted lipase that hydrolyzes host lipids (15–18), most research has focused on its potential role in promoting tissue invasion and pathogenesis (26). Here, we show that Geh has a physiological role in S. aureus membrane phospholipid homeostasis. Geh acts as a 1-position specific phospholipase A1 responsible for the formation of 2–12(S)-methyltetradecanoyl-*sn*-glycero-3-phospho-*sn*-1′-glycerol (a15-LPG) that is released into the extracellular environment.

## RESULTS

### Composition of the S. aureus LPG pool.

**FIG 1:**
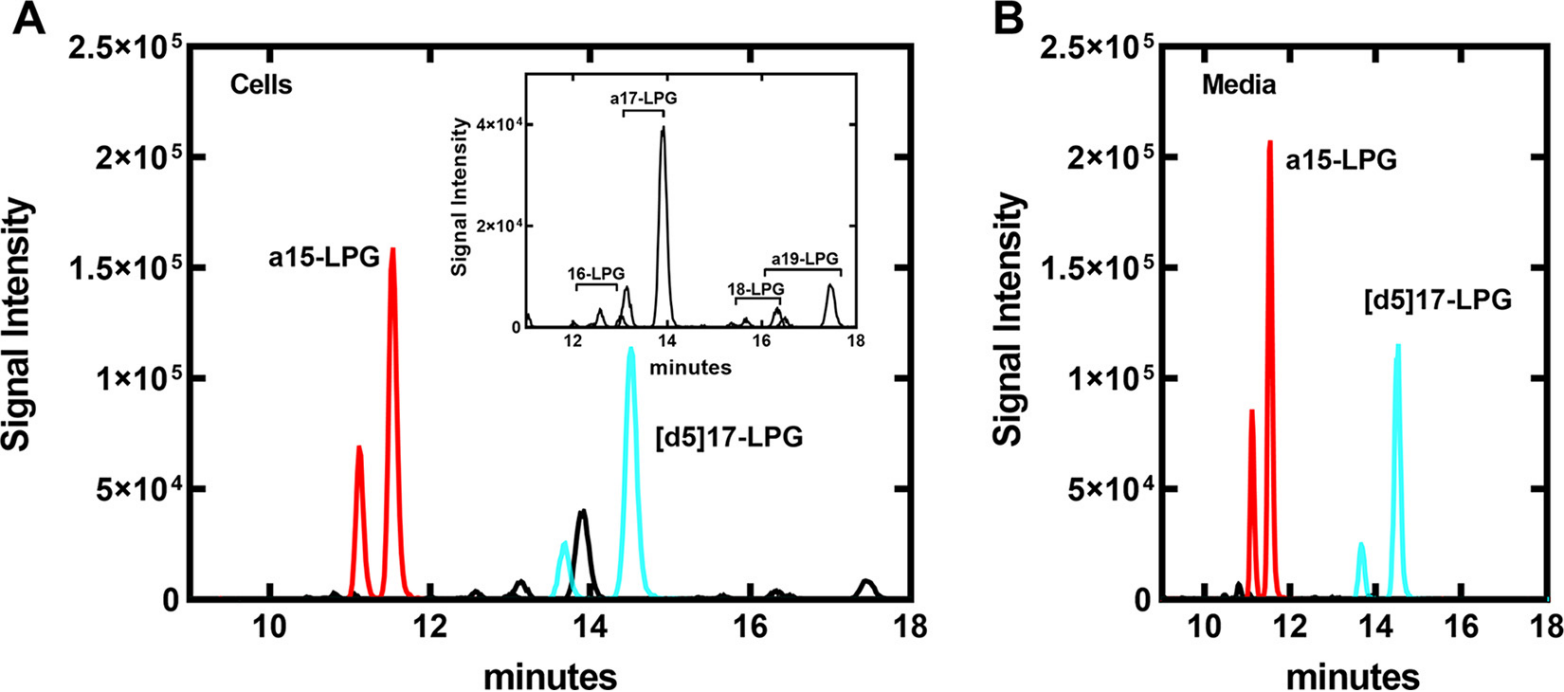
LPG molecular species composition of cells and media. Strain JE2 (wild type) was grown to an A_600_ of 4.0 and LPG was extracted from cells pelleted from 5 mL of media or from 1 mL of the cell supernatant passed through a 0.2 μm filter. LPG was extracted and the molecular species detected by LC-MS/MS. A [d5]17-LPG internal standard spiked into the samples. Red peaks are a15:0-LPG, black peaks are ≥C16-LPG, and cyan peaks are [d5]17-LPG. (A) LPG from cells. *Inset*, the region from 11 to 18 min of the gradient is zoomed in with the internal standard channel removed to clearly show the distribution of less abundant cellular ≥C16-LPG species. (B) LPG molecular species detected in cell-free media. Representative scans were selected from three or more independent experiments.

S. aureus PG has a strict positional distribution of fatty acids when grown in rich broth (27–29). The *sn*-2-position is dominated by 12(S)-methyltetradecanoic acid (a15), and the *sn*-1-position contains ≥16-carbon acyl chains (≥16) giving rise to the prototypical PG molecular species distribution annotated in Fig. S1. The cellular LPG pool composition was determined by LC/MS/MS (Fig. 1). The most abundant LPG was a15-LPG (Fig. 1A), which implies that it arises from the removal of the 1-position acyl chain of PG. The other LPGs contain acyl chains normally found in the 1-position of PG and would arise from the removal of the a15 from the 2-position. The composition of the ≥16-LPGs (Fig. 1A, *Inset*) reflects their relative abundance in the PG molecular species (Fig. S1). We detected substantial quantities of a15-LPG in S. aureus conditioned media, whereas the extracellular concentrations of the ≥16-LPGs were almost below detection (Fig. 1B). The growth media alone had undetectable a15-LPG (<0.6 pmol/mL). These data suggest that the bulk of the a15-LPG produced by S. aureus was released into the environment.

10.1128/msphere.00031-23.3 FIG S1 Annotated PG molecular species composition of S. aureus. The example shown is from strain AH1263 (wild-type) grown in rich broth. The number above the line is the total carbon number of the PG molecular species and the 1-position/2-position acyl chains are indicated below the line. The PG molecular species have ≥C16 acyl chains at the 1-position and a15 in the 2-position (red). Download FIG S1, TIF file, 0.5 MB. Copyright © 2023 Subramanian et al. 2023 Subramanian et al. https://creativecommons.org/licenses/by/4.0/ This content is distributed under the terms of the Creative Commons Attribution 4.0 International license.

**FIG 2:**
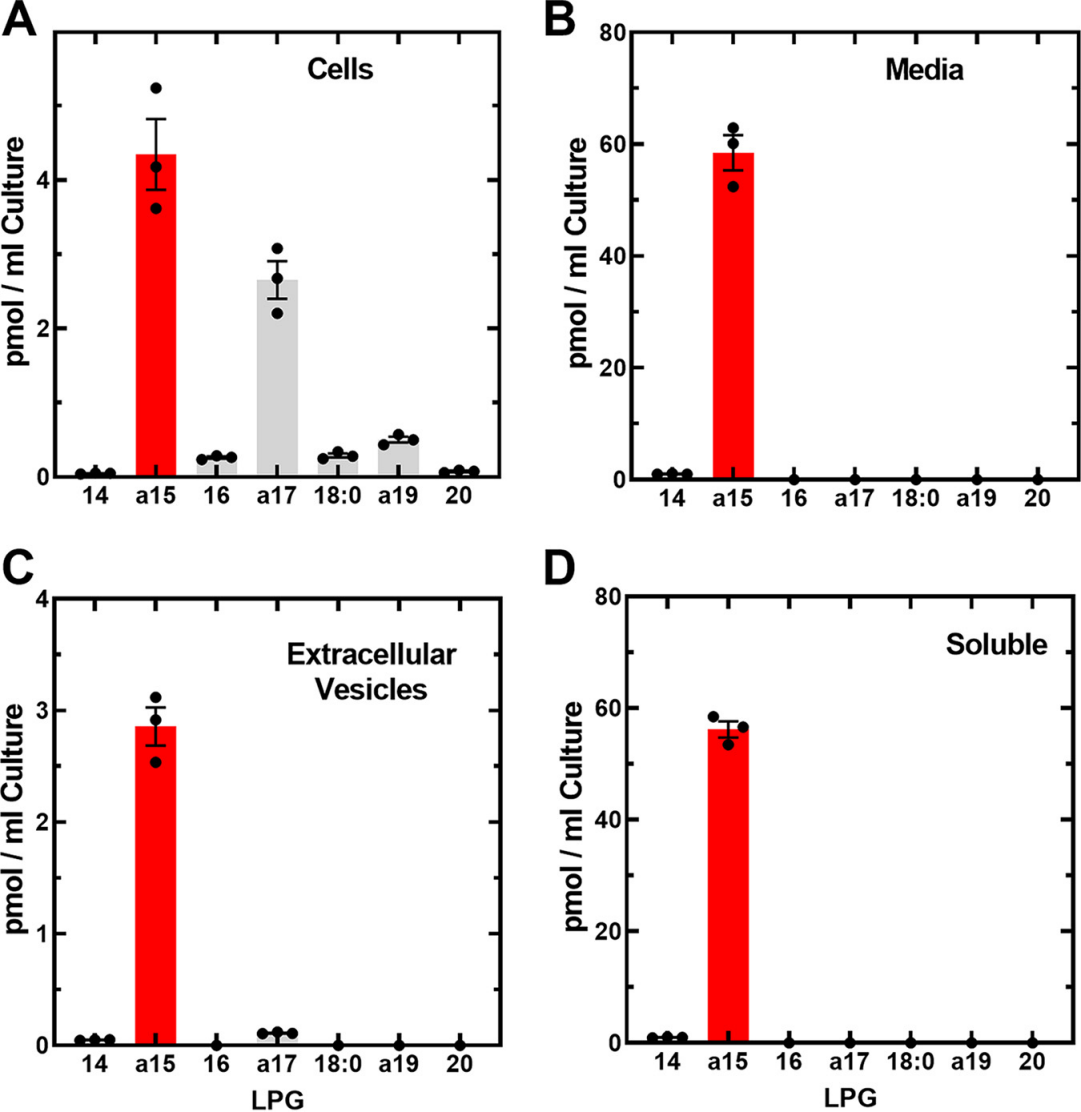
Localization of extracellular a15-LPG in the soluble fraction. Strain AH1263 (wild type) was grown in rich broth to an A_600_ of 4.0, and the cells separated from the media by centrifugation and the medium was filtered through a 0.2 μm filter. The media sample was then fractionated by ultracentrifugation to pellet extracellular vesicles and membrane fragments. LPG molecular species present in different fractions were quantified by LC-MS/MS in triplicate experiments using [d5]17:0-LPG as the internal standard. (A) LPG molecular species present in intact cells of strain AH1263. (B) LPG molecular species present in filtered media. (C) LPG molecular species present in the vesicle fraction of the media (Panel B) isolated by ultracentrifugation. (D) LPG composition of the soluble fraction of the media.

The abundance of the LPG molecular species in different fractions from 1 mL of cultured wild-type strain AH1263 cells were quantified using the [d5]17-LPG internal standard (Fig. 2). The cellular LPG pool was a mixture of a15-LPG and ≥16-LPG (Fig. 2A). The media samples were filtered through a 0.2 μm filter to eliminate intact cells, and a15-LPG was the dominate component of the media fraction (Fig. 2B). However, exosomes or other membrane fragments may pass through the filter. The filtered media sample was centrifuged a 100,000 × *g* for 60 min to pellet exosomes, and the pellet and supernatant fractions analyzed for LPG (Fig. 2). The ≥16-LPGs molecular species in the pellet fraction reflected the composition of the cells; however, a15-LPG was enriched relative to ≥16-LPGs in the media vesicle fraction (Fig. 2C) compared to the cells (Fig. 2A). Only a small fraction of extracellular a15-LPG was associated with the vesicle fraction (Fig. 2C and 2D). The bulk of the a15-LPG was recovered in the supernatant from the high-speed centrifugation, and this fraction contained only a minor contamination with 14-LPG (Fig. 2D). Lysophospholipids can partition into phospholipid bilayers but are also water soluble. For example, 14-LPG solubility is ~300 μM (30) so finding a15-LPG in the medium is consistent with the biophysical properties of the molecule.

Mass spectrometry alone cannot directly determine whether a 15-carbon LPG has a straight chain or has an *iso* or *anteiso* branch. We performed a mass tracing experiment using strain AH1263 grown in defined media where the normal concentrations of branched-chain amino acids were replaced with [^13^C_6_]isoleucine, [*d*_3_]leucine, and [^13^C_5_,^15^N]valine to impart specific mass tags into the derivative branched-chain fatty acids allowing the identification of their origin and structure (29). The *anteiso* odd-carbon acyl chains derived from Ile have a +5-mass tag, the *iso* odd-carbon acyl chains have a +3-mass tag derived from Leu and *iso* even carbon acyl chains have a +4-mass tag derived from Val (29). The 15-LPG species in both the cells (Fig. S2A) and the media (Fig. S2B) predominantly contained the +5-mass tag derived from Ile, meaning that it is a15. There was also a normal mass (unlabeled) 15-LPG peak that arises from 2-methylbutyryl-CoA derived from the *de novo* Ile biosynthetic pathway, and a smaller proportion of Leu-derived (+3) *iso* 15-carbon acyl chains. These data show that a15:0 is the predominant acyl chain in extracellular LPG.

10.1128/msphere.00031-23.4 FIG S2 Verification of a15-LPG structure. Strain AH1263 (wild-type) was grown in minimal media containing [^13^C_6_]isoleucine, [d3]leucine and [^13^C_5_,^15^N]valine. The major peak of 15:0-LPG was +5 (green) which would be derived from [^13^C_6_]isoleucine. The +3 15:0-LPG (red) would be derived from leucine. The unlabeled 15-LPG (black) was synthesized by the *de novo* pathway. Representative data from three independent experiments is shown. Download FIG S2, TIF file, 0.2 MB. Copyright © 2023 Subramanian et al. 2023 Subramanian et al. https://creativecommons.org/licenses/by/4.0/ This content is distributed under the terms of the Creative Commons Attribution 4.0 International license.

### Identification of Geh as the source of extracellular a15-LPG.

**FIG 3:**
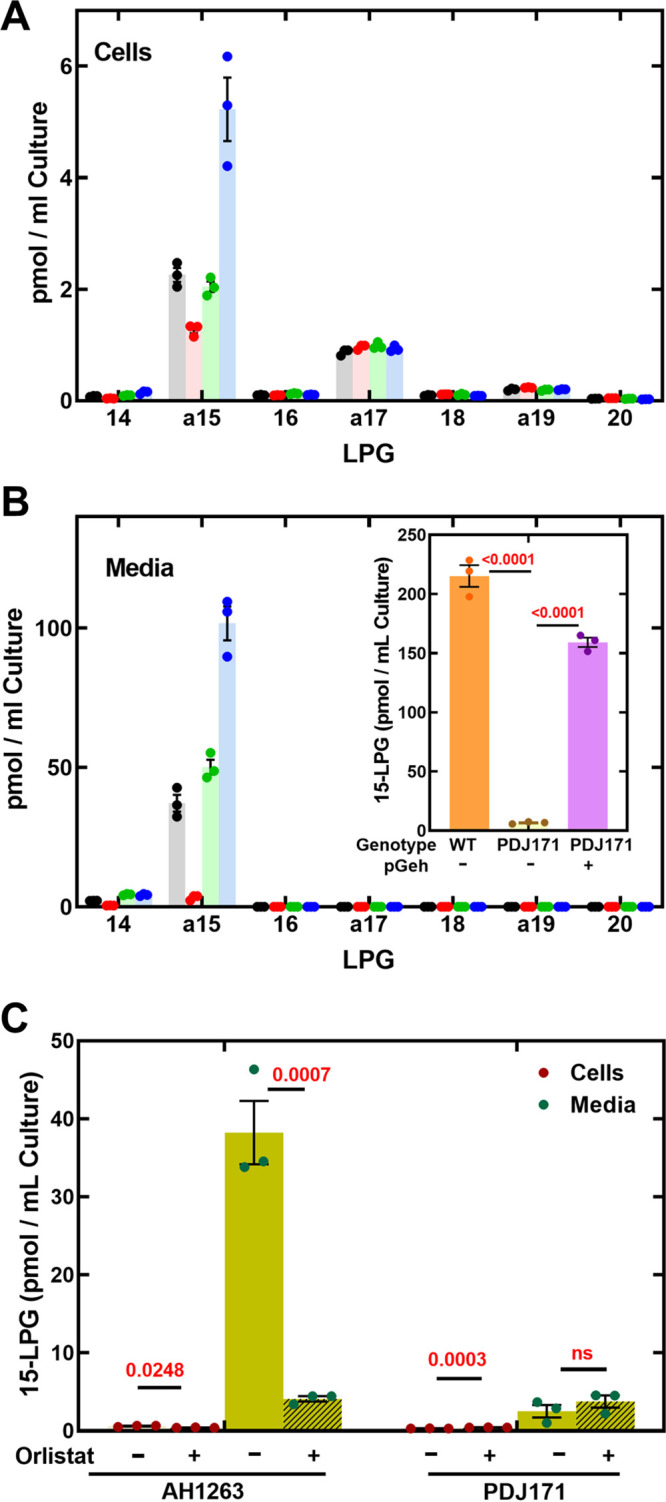
Identification of Geh as responsible for extracellular a15-LPG formation. The parent strain JE2 and three strains from the Nebraska transposon collection with inactivating insertions in each of three extracellular lipase genes (*lip*::ϕNΣ, *geh*::ϕNΣ and *SAUSA300_0641*::ϕNΣ) were grown to OD_600_ of 4 and the cells and media LPG molecular species quantified by LC-MS/MS using [d5]17:0-LPG as the internal standard. Strain JE2 (wild-type), gray; strain NE1175 (*geh*::ϕNΣ), pink; strain NE338 (*lip*::ϕNΣ), green; and strain NE104 (*SAUSA300_0641*::ϕNΣ), blue. (A) Composition of cellular LPG molecular species in the strain panel. (B) Composition LPG released into the filtered media. *Inset*, strains AH1263 and PDJ171 (Δ*geh*) were transformed with the empty pPJ480 vector and the production of extracellular a15-LPG compared to strain PDJ171 *(Δgeh*)/pGeh with the Geh expression vector. AH1263/pPJ480 (orange), PDJ171 *(Δgeh*)/pPJ480 (brown), and PDJ171 (Δ*geh*)/pGeh (purple). (C) Strains AH1263 and PDJ171 (Δ*geh*) were treated with 20 μg/mL Orlistat or a DMSO only control and LPG was extracted from cells and media at A_600_ 4.0. The amounts of a15-LPG was determined by LC-MS/MS in the DMSO-treated control cells (maroon) and media (green), or in samples treated with 20 μg/mL Orlistat (hatched bars). *P* values calculated by unpaired parametric *t* test are shown in red.

The appearance of extracellular a15-LPG suggested that an extracellular lipase/phospholipase may be involved. There are three major secreted lipases in S. aureus: a short-chain esterase/lipase (Lip or SAL1), glycerol ester hydrolase (Geh or SAL2), and (SAL3) (11, 31). The concentrations of extracellular a15-LPG produced by strains from the Nebraska transposon library (32) containing inactivating insertions in each of these lipase genes (Table S1) were compared to the parental strain JE2. There was no impact on the size or composition of the cellular ≥16-LPG pools in the three knockout strains compared to strain JE2 (Fig. 3A). The cellular a15-LPG concentration was reduced by half in strain NE338 (*geh*::ϕNΣ) but was not reduced in the other knockouts (Fig. 3A). Strain NE338 (*geh*::ϕNΣ) was significantly (*P < *0.001) deficient in the production of extracellular a15-LPG (Fig. 3B). The fact that a pool of cellular a15-LPG remained present in the *geh*::ϕNΣ strain means that there is also a Geh-independent source of cellular a15-LPG.

10.1128/msphere.00031-23.1 TABLE S1 Strains and plasmids used in this work. Download Table S1, DOCX file, 0.02 MB. Copyright © 2023 Subramanian et al. 2023 Subramanian et al. https://creativecommons.org/licenses/by/4.0/ This content is distributed under the terms of the Creative Commons Attribution 4.0 International license.

These results were validated by constructing strain PDJ171 (Δ*geh*), a derivative of wild-type strain AH1263 that was constructed to contain an unmarked deletion of the *geh* gene coding sequence (20). The production of extracellular a15-LPG was measured in strains AH1263/pPJ480, PDJ171 (Δ*geh*)/pPJ480 containing the empty vector, and strain PDJ171 (Δ*geh*)/pGeh programed to express *geh* driven by the *sarA* promoter (Table S1). This Δ*geh* strain was also severely deficient in the production of extracellular a15-LPG, which was restored by the plasmid-driven expression of the *geh* gene (Fig. 3B, *Inset*). Orlistat, a covalent inhibitor of pancreatic lipase, also blocks Geh activity (33). The treatment of strain AH1263 with 20 μg/mL Orlistat significantly reduced the formation of extracellular a15-LPG (Fig. 3C). Orlistat treatment also reduced the cellular concentration of a15-LPG (Fig. 3C) similar to the reductions in cellular a15-LPG noted in the Δ*geh* strain (Fig. 3A). These experiments show Geh is the most important source of extracellular a15-LPG.

### Geh biochemistry.

The *geh* gene encodes preproGeh (34) (Fig. S3A). The signal sequence (amino acids 1 to 37) is cleaved coincident with the export of the protein through the plasma membrane to form proGeh. Then, a proportion of proGeh is cleaved by aureolysin, a secreted metalloprotease, to produce mature Geh (mGeh) (23, 35) (Fig. S3A). Both proGeh and mGeh are abundant extracellular proteins (23), and in the prior experiments with cells, Geh refers to the mixture of proGeh and mGeh that normally arise in S. aureus from expression of the *geh* gene. Prior biochemical work with the two isoforms has not revealed a difference in their activities toward the substrates employed in the studies (23, 24). Geh is considered a nonspecific lipase (36) that at sufficient concentrations of enzyme and substrate is capable of hydrolyzing a wide array of long-chain esters, including p-nitrophenyl esters of butyrate (33) or palmitate (21), triacylglycerols (36), and diacyl-glycerol-modified lipoproteins (21). However, for Geh to be responsible for a15-LPG formation, its physiological function would be to act as a 1-position-specific phospholipase A1, an activity that has not been studied in Geh.

10.1128/msphere.00031-23.5 FIG S3 Structure and purification of proGeh and mGeh. (A) A schematic diagram of Geh. The primary translational product is preproGeh that is cleaved to proGeh concomitant with the transport of Geh out of the cell. Extracellular proGeh is cleaved by the aureolysin protease (*) to produce mGeh. proGeh and mGeh were expressed in E. coli as a carboxy-terminal His-tagged proteins and purified by Ni^2+^ affinity and gel filtration chromatography. (B) Size exclusion chromatography of proGeh showing it is primarily a dimer. *Left inset*, calibration of the gel filtration column with standards. *Right inset*, SDS gel electrophoresis illustrating the purity of proGeh. (C) Size exclusion chromatography of mGeh showing that both monomers and dimers are present. *Left inset*, calibration of the gel filtration column with standards. *Right inset*, SDS gel electrophoresis illustrating the purity of mGeh. Download FIG S3, TIF file, 7.7 MB. Copyright © 2023 Subramanian et al. 2023 Subramanian et al. https://creativecommons.org/licenses/by/4.0/ This content is distributed under the terms of the Creative Commons Attribution 4.0 International license.

**FIG 4:**
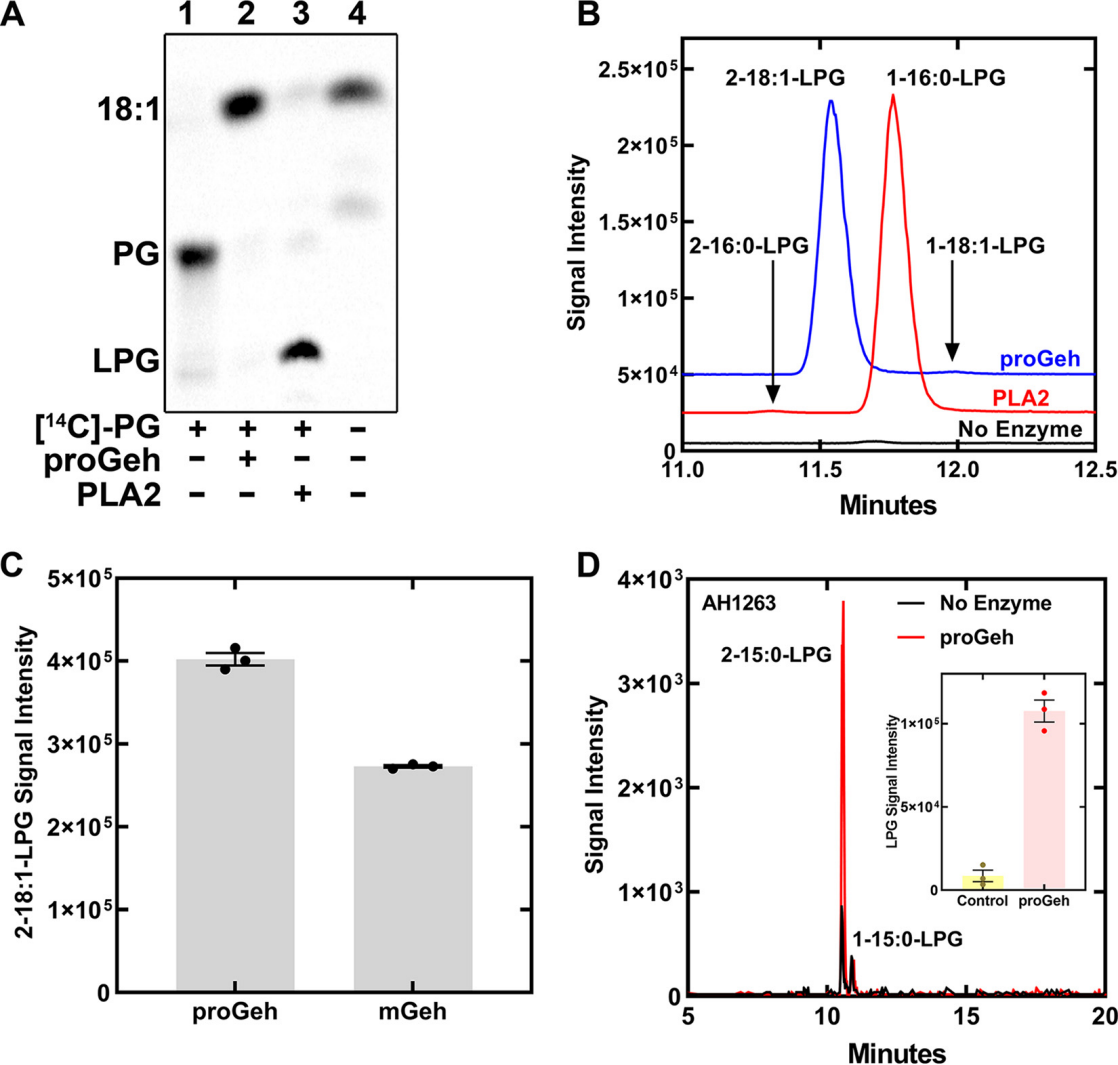
Positional specificity of Geh hydrolysis of PG. (A) [^14^C]PG labeled in the 1-position was isolated from S. aureus metabolically labeled with [^14^C]oleate. [^14^C]PG was digested with either proGeh or bee venom PLA2 and the products separated by thin-layer chromatography and visualized using a PhosphorImager. Lane 1, [^14^C]PG, lane 2, proGeh digest of [^14^C]PG, lane 3, PLA2 digest of [^14^C]PG, lane 4, [^14^C]oleate standard. (B) Liposomes composed of 1-palmitoyl-2-oleoyl-PG were digested with either proGeh or PLA2 at pH 6.0, and the LPG products analyzed by LC-MS/MS. The blue line shows the 2- oleoyl-LPG product of the proGeh reaction and the red line shows the 1- palmitoyl-LPG product of the PLA2 reaction. The elution positions of the trace amounts of 1-oleoyl-LPG and 2-palmitoyl-LPG detected are indicated with arrows. (C) The formation of 2-oleoyl-LPG by proGeh and mGeh were compared using liposomal 1-palmitoyl-2-oleoyl-PG as the substrate. (D) The hydrolysis of S. aureus phospholipid liposomes by proGeh analyzed by LC-MS/MS. Red trace, proGeh hydrolysis products. Black trace, no proGeh, to control for the a15-LPG present in the cellular phospholipid extract. Inset: the experiments were performed in triplicate.

Both proGeh with an amino-terminal His tag (Fig. S3B) and mGeh with a carboxy-terminal His tag (Fig. S3C) were expressed in E. coli and purified by Ni^2+^ affinity and gel filtration chromatography. The proGeh was primarily a dimer, whereas mGeh was a mixture of monomers and dimers based on gel filtration chromatography (Fig. S3B and S3C). Lipid metabolic enzymes exhibit stereo-selectivity for utilizing either the 1- or 2-positions of the *sn*-glycerol-3-phosphate backbone. Bee venom phospholipase A2 (PLA2) was used as a control for a phospholipase that selectively hydrolyzes acyl chains at the 2-position of phospholipids. S. aureus was labeled with [1-^14^C]oleate and the PG fraction was isolated by solid-phase chromatography. Oleate is selectively incorporated into the 1-position of PG (27, 37), therefore [^14^C]fatty acid is formed by a phospholipase A1 reaction and [^14^C]LPG is formed by a phospholipase A2 reaction. The [^14^C]PG was digested with either proGeh or PLA2 and the products separated by thin-layer chromatography (Fig. 4A). The product of the Geh digest was [^14^C]fatty acid indicating hydrolysis of the 1-position acyl chain, and [^14^C]LPG was the PLA2 product indicating hydrolysis at the 2-position acyl chain.

Product formation by proGeh and PLA2 was examined further using 1-palmitoyl-2-oleoyl-PG (1-16:0-2-18:1-PG) as the substrate at pH 6.0 followed by immediate characterization of the products by LC-MS/MS. The 1- and 2-position LPGs are separated from each other in the liquid chromatography fractionation step in the LC-MS/MS workflow (Fig. 1) (38). The product of the proGeh reaction was 2–18:1-LPG and the PLA2 product was 1–16:0-LPG (Fig. 4B) showing that proGeh is a 1-position-specific phospholipase A1 and that PLA2 selectively hydrolyzes the 2-position. The acyl chains of lysophospholipids undergo spontaneous migration between the 1- and 2-positions of the glycerol backbone, and at equilibrium they reach about 90% 1-position (39–41). Suppressing this acyl migration during the analytical workflow was important to obtaining the data in Fig. 4B. Acyl chain migration is base catalyzed (39–41). Performing Geh assays at pH 8.5 followed by the immediate injection and analysis of the products resulted in detecting a mixture of 1- and 2–18:1-LPGs (Fig. S5A). If the sample was analyzed 12 h later, the 1–18:1-LPG isomer was the predominant form detected (Fig. S5B). When the Geh reaction was performed at pH 6.0, 2-18:1-LPG was the major product with only a minor amount 1-acyl-LPG detected (Fig. S5C). At this pH, only about 10% of the sample isomerized to the 1-position after 12 h (Fig. S5D). Thus, performing Geh assays at pH 6.0 greatly minimizes acyl chain migration, and coupled with an immediate analysis of the products by LC-MS/MS, provides a clear indication of the enzyme’s positional specificity. In practice, samples are usually extracted, dried, and resuspended in solvent. It is common for sample preparation to take a day and for the samples to sit in the autosampler for 12–24 h before injection when a series of experiments with multiple samples are being analyzed. It is not possible to prevent acyl chain migration in these circumstances resulting in the detection of a mixture of isomers in most experiments. We also tested if both proGeh and mGeh were active in the formation of 2-acyl-LPG and found that both enzyme forms had robust phospholipase A1 activity, although mGeh was slightly less active under these conditions (Fig. 4C). The hydrolysis of a S. aureus lipid extract with proGeh at pH 6.0 with immediate injection showed that 2-a15-LPG was the only LPG detected (Fig. 4D). S. aureus plasma membranes were isolated by lysostaphin treatment, sonication and ultracentrifugation (42). The isolated membranes incubated with proGeh generated large quantities of a15-LPG (Fig. S4) illustrating that the membrane PG is a substrate for Geh. These data show that both proGeh and mGeh are phospholipase A1 enzymes and that the biologically relevant Geh product is 2-a15-LPG. This biologically relevant product undergoes acyl chain migration during sample workup and analysis to give rise to the two peaks of a15-LPG observed in the chromatograms (Fig. 1).

10.1128/msphere.00031-23.6 FIG S4 Geh hydrolysis of S. aureus membrane PG. Membranes from S. aureus strain NE1360 (*mprF*::ϕNΣ) were isolated by lysostaphin treatment, hypotonic lysis and ultracentrifugation. The membrane fraction (180 μg protein) was then incubated at 37°C for 1 h with proGeh (60 μM) and the formation of a15-LPG determined by LC-MS/MS. Red trace, membranes alone; blue trace, with proGeh. Download FIG S4, TIF file, 0.10 MB. Copyright © 2023 Subramanian et al. 2023 Subramanian et al. https://creativecommons.org/licenses/by/4.0/ This content is distributed under the terms of the Creative Commons Attribution 4.0 International license.

10.1128/msphere.00031-23.7 FIG S5 The pH dependence of positional isomerization in lysophospholipids. Reactions were carried out using Geh with 1-16:0-2-18:1-PG as the substrate for 15 min. The samples were either immediately injected into the LC-MS/MS system without concentration or workup, or left to stand for 12 at room temperature. (A) proGeh reaction at pH 8.5 followed by the immediate injection of the sample onto LC-MS/MS system. (B) proGeh reaction at pH 8.5 followed by the injection of the sample onto LC-MS/MS system after 12 h at room temperature. (C) proGeh reaction at pH 6.0 followed by the immediate injection of the sample onto LC-MS/MS system. (D) proGeh reaction at pH 6.0 followed by the injection of the sample onto LC-MS/MS system after 12 h at room temperature. Download FIG S5, TIF file, 0.1 MB. Copyright © 2023 Subramanian et al. 2023 Subramanian et al. https://creativecommons.org/licenses/by/4.0/ This content is distributed under the terms of the Creative Commons Attribution 4.0 International license.

## DISCUSSION

**FIG 5:**
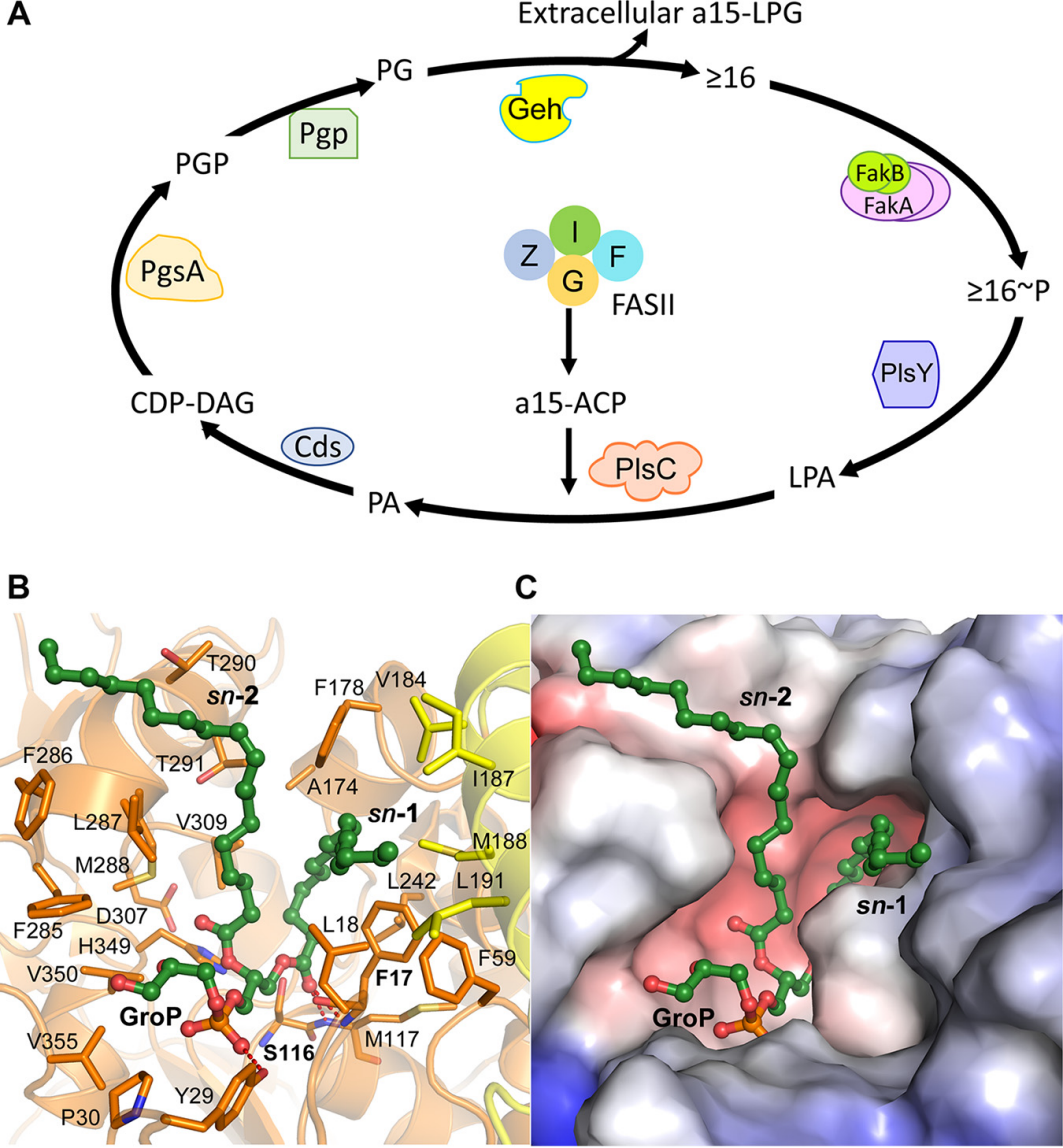
Role of Geh as a phospholipase A1 in membrane PG turnover. (A) Geh cleaves the one position fatty acid of PG, the major membrane phospholipid of S. aureus. The bulk of the a15-LPG product is released from the cell. The fatty acid product (≥16) is activated by the fatty acid kinase system that consists of the kinase domain protein (FakA) that phosphorylates the fatty acid bound to the fatty acid binding protein (FakB). The resulting acyl-phosphate (≥16~P) is a substrate for PlsY, the glycerol-phosphate acyltransferase that initiates PG synthesis. PlsC specifically utilizes a15-ACP arising from the fatty acid elongation cycle (FASII). Phospatidic acid (PA) is converted to PG in 3 steps. The cycle regenerates the same spectrum of PG molecular species that Geh hydrolyzed, thus maintaining membrane compositional homeostasis. (B) 1-Palmitoyl-2-oleoyl-PG docked into the active site cavity of Geh (PDB 6KSM) with the carbonyl of the *sn*-1 acyl chain positioned in the oxyanion hole created by Ser116 and Phe17. An initial docking solution was obtained using the SwissDock server, and energy minimization of the ligand was performed using Molecular Operating Environment (MOE) (2018.01; Chemical Computing Group). The *sn*-1 and *sn*-2 position fatty acids extend into two hydrophobic substrate channels in Geh, and the PG headgroup (GroP) lies in the more polar cavity. (C) Surface representation of the Geh substrate cavity with PG docked in the Geh active site. Electrostatic potential surface of Geh was drawn using the Adaptive Poisson-Boltzmann Solver (APBS) package within PyMOL. The electrostatic potential ranges from −5 to 5 kT/e. Red is negative, blue is positive and white is neutral/hydrophobic potential. All structures were rendered with PyMOL (version 2.5.1, Schrödinger, LLC).

The discovery that Geh is the major contributor to the formation and release of a15-LPG in S. aureus reveals an unappreciated physiological role for Geh acting as a phospholipase A1 in the degradation of S. aureus membrane PG (Fig. 5A). It is known that the acyl chains of S. aureus PG turnover and that the resulting fatty acids are metabolized by the fatty acid kinase system (Fig. 5A). Fatty acid kinase consists of a kinase domain protein (FakA) that phosphorylates a fatty acid bound to a fatty acid binding protein (FakB) (37, 43–45). Fatty acid kinase knockout strains have an elevated cellular fatty acid pool that consists of ≥16 fatty acids derived from the 1-position of PG (46). These data suggested that the a15 acyl chain in the 2-position of PG is used for another purpose and is not reincorporated into the membrane. This work identifies the Geh-dependent release of a15-LPG into the environment, leaving the fatty acid product associated with the cells, as one source of cellular ≥16 fatty acid arising from PG turnover. The fatty acid kinase system activates these fatty acids to acyl-phosphates and they are reintroduced into the phospholipid biosynthetic pathway at the PlsY step (Fig. 5A). Geh is not the only source for a15-LPG, which may arise from N-terminal acylation of lipoproteins by LnsAB (10), other cellular biosynthetic processes or other α/β-hydrolases. PlsC is highly specific for a15-ACP arising from the fatty acid elongation cycle. Therefore, this PG turnover cycle maintains membrane compositional homeostasis by regenerating the same spectrum of PG molecular species that were degraded by Geh to initiate the turnover cycle (Fig. 5A).

The role(s) for extracellular a15-LPG in S. aureus biology remain to be elucidated. Bacillus subtilis also releases a15-LPG into the environment (47). These investigators named the molecule bacilysocin and proposed it may function as an antibiotic against certain fungi. The fact a15-LPG release is common to these related bacteria that inhabit different environments suggests there may be a shared physiological function for the process. PG hydrolysis by extracellular Geh would create a15-LPG on the outer leaflet of the PG bilayer with the potential to introduce positive curvature to the membrane. The role of bilayer curvature introduced by asymmetric lysophospholipid generation in the formation of budding vesicles in other systems (48, 49) suggests that Geh hydrolysis of the outer leaflet of the PG bilayer may function similarly to facilitate formation and budding of exosomes (50–52). The a15-LPG content of the media vesicle fraction shows it is enriched in a15-LPG compared to the cellular membrane; however, more research is needed to determine if this correlation is functionally relevant. The bulk of the extracellular a15-LPG is soluble, and a signaling role for a15-LPG in S. aureus is possible. Lysophospholipids are established second messengers in mammalian biology (1) suggesting that LPG release may have a role in either S. aureus signaling and/or virulence by altering host responses. Although the role of LPG in signaling is less studied than other lysophospholipids (1), it is known that LPG specifically triggers calcium mobilization and phospholipase C activation in cell lines (53), is a competitive inhibitor of the LPA receptor in Jurkat T cells (54), and stimulates chemotactic migration of natural killer cells (55, 56). Validating the potential targets for a15-LPG action in modulating host responses will be important to understanding the role of this molecule in pathogenesis.

PG modeled into the active site of Geh using the Geh•Orlistat complex structure provides insight into Geh phospholipase A1 activity (Fig. 5B). The *sn*-2 chain of PG lies in the hydrophobic cavity occupied by the Orlistat β-side chain and the *sn*-1 acyl chain sits in the space occupied by the α-side chain of Orlistat (Fig. S6A). The glycerol head group sits in the cavity occupied by the shorter and more polar amino ester side chain of Orlistat (Fig. 5B and S6A). The *sn*-1 ester is positioned adjacent to the active site serine with the carboxyl group sitting in the oxyanion hole formed by the backbone amide protons of Ser116 and Phe17 (Fig. 5B). The fixed orientation of the two acyl chains in the active site cavity (Fig. 5C) explain the selectivity of Geh for the *sn*-1 position of PG. The Geh homolog from *S. hyicus* (Shl) has robust lipase and phospholipase A1 activity (22, 57). The similarities between the crystal structures of *S. hyicus* Shl and Geh are striking (Fig. S6B). The only region where the two proteins diverge is in the relative orientation of the lid, which is a dynamic region consisting of two α-helices that move to allow substrate entry to and exit from the active site (58). Modeling dioctanoyl-phosphatidylcholine into the Shl active site yields similar conclusions concerning the positioning of the *sn*-1 ester for hydrolysis (59). The Lip lipase of S. aureus, which does not have a homolog in *S. hyicus*, is most active on short-chain triacylglycerols and lacks phospholipase A1 activity (57). Understanding the structural differences between Geh and Lip that confer the substrate selectivity of the two proteins awaits crystal structures of Lip.

10.1128/msphere.00031-23.8 FIG S6 Geh structure and PG binding. (A) Overlay of the structure of Orlistat (pink) bound to Geh (PDB ID: 6KSM) overlaid with the docked structure of PG (green). The *sn*-2 chain of PG lies in the cavity occupied by the Orlistat β-side chain and the *sn*-1 acyl chain sits in the space occupied by the Orlistat α-side chain. The PG head group (GroP) sits in the cavity occupied by the more polar amino ester side chain of Orlistat. S116 is the active site serine. (B) Comparison of the crystal structures of *S. hyicus* lipase/phospholipase A1 (Shl; PDB ID: 2HIH) (grey) and S. aureus Geh (PDB ID: 6KSM) (orange). Ca^2+^ (green ball) has a structural role and is located at the same position in both enzymes. The position of Orlistat (pink) in the Geh structure is shown. The only region that is slightly different in Shl and Geh is the lid domain (α7 + α8; yellow) that opens and closes to allow substrate entry and exit from the active site. The R.M.S.D. difference between the Shl and Geh structures excluding the lid region are 1.101 Å and the full molecule R.M.S.D. is 1.562 Å. Download FIG S6, TIF file, 1.1 MB. Copyright © 2023 Subramanian et al. 2023 Subramanian et al. https://creativecommons.org/licenses/by/4.0/ This content is distributed under the terms of the Creative Commons Attribution 4.0 International license.

## MATERIALS AND METHODS

### Materials.

All chemicals and reagents were reagent grade or better. [^13^C_6_]Isoleucine, [*d*_3_]leucine, [^13^C_5_,^15^N]valine were purchased from Cambridge Isotope Laboratories, Inc. DSC-NH_2_ SPE columns (Supelco, Sigma-Aldrich), Bee venom PLA_2_ (P9297-Sigma), Orlistat (O4169-Sigma), [1-^14^C]oleate (specific activity 59 mCi/mmol) (Perkin Elmer).

### Bacterial strains and media.

S. aureus strains are listed in Table S1. Defined media components and other reagents were purchased from Millipore Sigma or Thermo Fisher Scientific. All mass spectrometry reagents are HPLC grade or better. The defined media used in this work consists of M9 salts supplemented with glucose, amino acids and other nutrients exactly as described (29). Defined media consisted of M9 salts, 1 mM MgSO_4_, 0.1 mM CaCl_2_, 15 μM vitamin B_1_, 32 μM vitamin B3, 0.4% glucose, 0.1 mg/L biotin, 2 mg/L pantothenic acid, 10 μM FeCl_2_, 6 mg/L citrate, 10 mg/L MnCl_2_, 4 mg/L l-tryptophan, 0.1 mg/L l-lipoic acid; and the amino acid concentrations were the same as in RPMI 1640 media (Sigma-Aldrich). S. aureus strains were routinely grown in rich broth (10 g tryptone, 5 g yeast extract, 5 g NaCl per L). Bacterial strains were grown in 15 mL culture tubes containing 3 mL rich broth overnight at 37°C with shaking at 200 rpm. The bacteria were subcultured in 30 mL culture flasks containing 10 mL fresh rich broth at OD_600_ 0.05 and grown to OD_600_ 4.0 at 37°C with shaking at 200 rpm.

### Molecular biology.

The *geh* gene was amplified by PCR using primers designed for Gibson Assembly cloning into NdeI and HindIII digested pET28a to obtain pPJ628 with a carboxy-terminal His tag used for E. coli expression and purification of proGeh and mGeh. These His-tagged constructs were then cloned into pPJ480 at the NcoI and HindIII sites to derive plasmid pPJ630 for expression in S. aureus driven by the *sarA* promoter (20). Plasmids were rendered transformable into normal S. aureus strains by passing them through strain RN4220.

### LPG extraction and isoleucine labeling.

Strains AH1263, PDJ171 (Δ*geh*) (20), JE2, NE338 (*SAUSA300_2603*::ϕNΣ), NE1175 (*SAUSA300_0320*::ϕNΣ) or NE104 (*SAUSA300_0641*::ϕNΣ) were grown in rich broth to a A_600_ of 4.0. LPG was extracted from 5 mL of cells or 1 mL of supernatant from 0.2 μm filtered media. The cells were resuspended in 0.5 mL water and 0.5 mL of cold methanol containing 1% acetic acid was added. To the 1 mL of filtered media, 1 mL of cold methanol containing 1% acetic acid was added. Samples were incubated on ice for 10 min and centrifuged at 20,000 × *g* for 20 min. Supernatants were dried in a speed vac concentrator, resuspended in 80% methanol and 500 picograms of [d5]17-LPG was added. For complementation experiments, strain AH1263 containing the vector pPJ480, strain PDJ171 (Δ*geh*) containing either the pPJ480 or pPJ630 vectors were grown in rich broth plus 10 μg/mL chloramphenicol to an A_600_ of 4.0. LPG was extracted from cells and media as described above. For heavy labeling of LPG, AH1263 cells were grown on defined media plates and grown overnight in defined media. The next morning, cells were washed with media containing no isoleucine, leucine or valine and then inoculated into defined media containing 0.5 μg/mL [^13^C_6_]isoleucine, 0.2 μg/mL [^13^C_5_,^15^N]valine and 0.5 μg/mL of [d3]leucine at an OD_600_ of 0.05, and then grown to an A_600_ of 1.0. LPG was extracted from cells and media as described above. AH1263 and PDJ171 cells were treated with 20 μg/mL of Orlistat and grown to a final A_600_ of 4.0. LPG was extracted from cells and media as described above.

### LPG mass spectrometry.

LPG was analyzed using a Shimadzu Prominence UFLC attached to a QTrap 4500 equipped with a Turbo V ion source (Sciex). Samples were injected onto an Acquity UPLC HSS C18, 2.5 μm, 3.0 × 150 mm column at 30°C (Waters) using a flow rate of 0.2 mL/min. Solvent A was 5 mM ammonium acetate + 0.1% formic acid, and Solvent B was 95% methanol + 5 mM ammonium acetate + 0.1% formic acid. The HPLC program was the following: starting solvent mixture of 35% A/65% B, 0 to 1 min isocratic with 65% B; 1 to 3 min linear gradient to 100% B; 3 to 30 min isocratic with 100% B; 30 to 32 min linear gradient to 65% B; 32 to 35 min isocratic with 65% B. The QTrap 4500 was operated in the negative mode, and the ion source parameters were: ion spray voltage, -4500 V; curtain gas, 30 lb/in^2^; temperature, 500°C; collision gas, medium; ion source gas 1, 20 lb/in^2^; ion source gas 2, 35 lb/in^2^; decluttering potential, -80 V; and collision energy, -30 V. The multiple reaction monitoring transitions for LPG species are listed in Table S2. [d5]17-LPG was used as the internal standard. The system was controlled by the Analyst software (Sciex) and analyzed with MultiQuan 3.0.2 software (Sciex). Data used in the figures was output using the Gaussian smoothing routine in the Analyst software (Sciex). Peaks corresponding to individual LPG species were quantified relative to the internal standard. The lower limit of LPG detection based on a standard curve using 16-LPG was 5 femtomoles.

10.1128/msphere.00031-23.2 TABLE S2 Mass transitions used to detect LPG molecular species by LC-MS/MS. Download Table S2, DOCX file, 0.01 MB. Copyright © 2023 Subramanian et al. 2023 Subramanian et al. https://creativecommons.org/licenses/by/4.0/ This content is distributed under the terms of the Creative Commons Attribution 4.0 International license.

### Purification of Geh and size exclusion chromatography.

pPJ628 (proGeh) and pPJ650 (mGeh) expression vectors were transformed into BL21(DE3) cells and grown in rich broth to an OD_600_ of 0.7 at 37°C with shaking at 200 rpm. The culture was cooled to 16°C and the cells were induced with 1 mM IPTG overnight with shaking at 200 rpm. Cells were harvested and resuspended in 20 mM Tris (pH 8.0), 200 mM NaCl, 10 mM imidazole. Cells were broken by two passages through a cell disruptor and centrifuged at 20,000 g for 45 min. Geh was purified from the supernatant using a Ni-NTA column by washing with 20 column volumes of each 20 mM Tris (pH 8.0), 200 mM NaCl containing 10 mM imidazole or 20 mM imidazole or 40 mM Imidazole. Geh was eluted with 20 mM Tris (pH 8.0), 200 mM NaCl, 250 mM imidazole. The eluant was further purified by gel filtration chromatography using a HiLoad 16/600 Superdex 200 column (Cytiva Life Sciences) equilibrated with 200 mM NaCl, 20 mM Tris, pH 7.5. The molecular weight was estimated by analyzing the peak size on a HiLoad 13/300 Super 200 analytical column (Cytiva Life Sciences). A standard curve was generated using thyroglobulin (669 kDa), ferritin (440 kDa), aldolase (158 kDa), conalbumin (75 kDa), ovalbumin (44 kDa), RNase A (13.7 kDa), and aprotinin (6.5 kDa).

### Geh and PLA2 biochemistry.

In vitro activity of Geh and PLA2 was analyzed by mass spectrometry. Liposomes for Geh assays were prepared with 1-palmitoyl-2-oleoyl-*sn*-glycero-3-phospho-(1′-*rac*-glycerol) by drying the lipid under N_2_ and hydrating with 50 mM Tris (pH 8.0) for 2 h at 37°C. Hydrated lipids were sonicated for 2 min twice in a water bath sonicator and then passed 30 times through an Avanti Mini Extruder with a 100 nm membrane. A 50 μL assay reaction mixture containing 100 mM bis-Tris (pH 6.0), 150 mM NaCl, 1 μM PG liposomes and 500 nM Geh was incubated at room temperature for 15 min and 50 μL of methanol containing 1% acetic acid was added to stop the reaction. The rection was centrifuged at 20,000 × *g* for 20 min and the samples were analyzed by LC-MS/MS. For PLA2 assay, a 50 μL assay reaction mixture containing 100 mM bis-Tris (pH 6.0), 50 mM CaCl_2_, 1 μM PG liposomes, and 0.5 μg PLA2 was incubated at room temperature for 1 h, and 50 μL of methanol containing 1% acetic acid was added to stop the reaction. The reaction was centrifuged at 20,000 × *g* for 20 min and the samples were analyzed by LC-MS/MS.

The [^14^C]PG substate was isolated from strain NE1360 (*mprf*::ϕNΣ) labeled with 20 μM [1-^14^C]oleate (specific activity 59 mCi/mmol) for 2 h. Lipids were extracted using Bligh-Dyer method (60), dried under N_2_ and resuspended in 1 mL chloroform:methanol (1:1). [^14^C]PG was purified on a DSC-NH_2_ SPE column equilibrated with hexane. The column was washed with chloroform:isopropanol (2/1) and then with ether:2% acetic acid to elute fatty acids. [^14^C]PG was eluted with chloroform:methanol:0.8 M sodium acetate (60:30:4.5). The eluate was dried under nitrogen and extracted twice with chloroform: water (1:1). [^14^C]PG (1.5 × 10^6^ dpm) was dried, hydrated with 50 mM Tris (pH 8.0) and liposomes were prepared as described above. Geh assays contained 50 mM Tris (pH 8.0), 150 mM NaCl, 35,000 dpm [^14^C]PG liposomes and 50 μM Geh in a final volume of 20 μL, and were incubated at 37°C for 30 min. A 20 μL PLA_2_ assay reaction mixture containing 50 mM Tris (pH 8.0), 1 mM CaCl_2_, 35,000 dpm [^14^C]PG liposomes and 0.4 μg PLA2 was incubated at room temperature for 30 min. A 15 μL aliquot of each reaction was spotted on a Silica gel H thin-layer chromatography plate developed with ethanol:chloroform:triethylamine:water (34:30:35:6.5); (vol/vol/vol/v) plus 1 mM EDTA. The thin-layer plate was exposed to a Phosphor screen and scanned by Typhoon PhosphoImager (Perkin Elmer). Membranes were prepared strain NE1360 by treating the cells with lysostaphin, followed by hypotonic lysis with sonication, and the membranes were collected by ultracentrifugation as described by Oku et al. (42).

### Docking PG in the Geh active site.

The 1-palmitoyl-2-oleoyl-PG substrate was docked with Geh by using SwissDock server (61, 62). For docking calculation, the ligand was energy minimized with Molecular Operating Environment (MOE) (2018.01; Chemical Computing Group). Geh monomer protein structure was generated from Geh-Orlistat complex structure (PDB 6KSM). All solvent molecules and ligands except calcium and zinc ions were removed from the structure and Ser116 was mutated to Ala. After completion of docking processes, the obtained one of the poses was manually adjusted so that the carbonyl oxygen atom of cleavage site was located in the oxyanion hole. The fitted ligand and Geh (Ser116) structures were imported into MOE and prepared using QuickPrep and the ligand was minimized energy with carbonyl oxygen atom fixed in the oxyanion hole.

### Statistics.

Statistical tests were performed using GraphPad Prism software version 9.1.2 (https://www.graphpad.com/scientific-software/prism/).

### Ethics statement.

All work with S. aureus described herein were done in accordance with protocols approved by the Institutional Biosafety Committees at St. Jude Children's Research Hospital (SJCRH).
