# Supplementary figures and images for: The Phospholipase A1 Activity of Glycerol Ester Hydrolase (Geh) Is Responsible for Extracellular 2-12(S)-Methyltetradecanoyl-Lysophosphatidylglycerol Production in Staphylococcus aureus
Source: mSphere. 2023 Mar 28;8(2):e00031-23. doi: 10.1128/msphere.00031-23 (PMC10117073; doi:10.1128/msphere.00031-23)

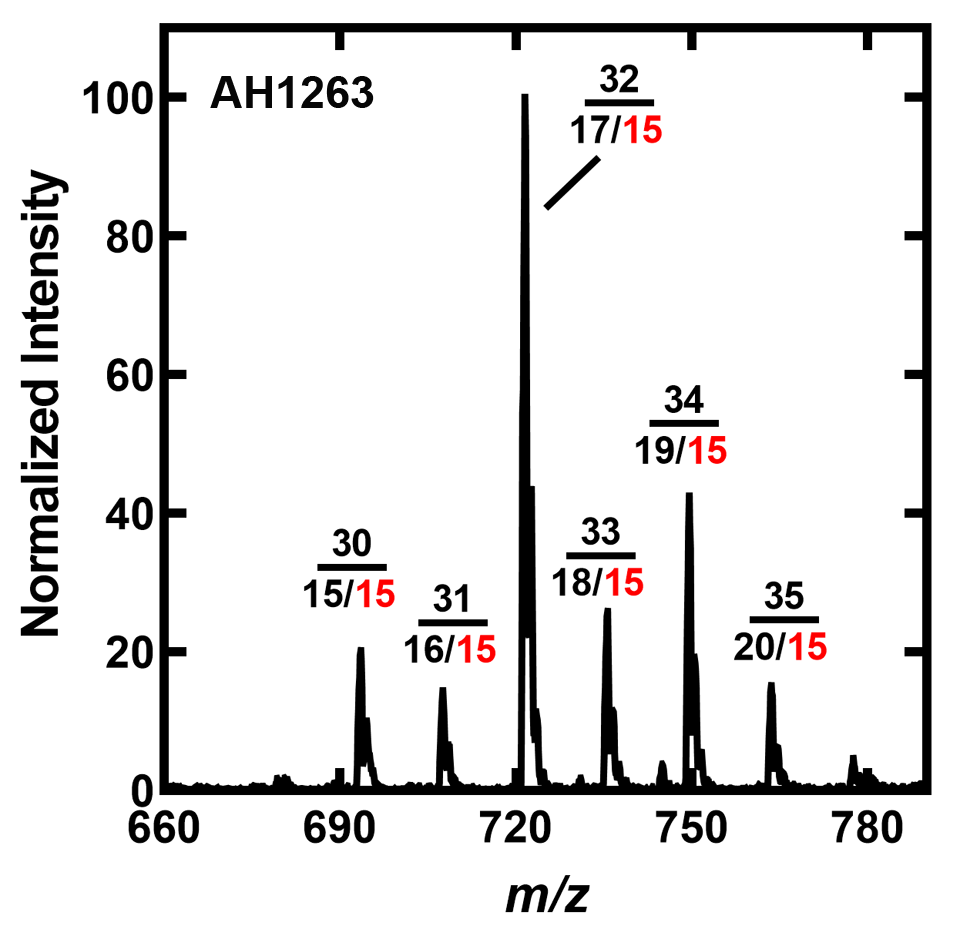

Supplement: FIG S1 [file msphere.00031-23-s0003.tif]

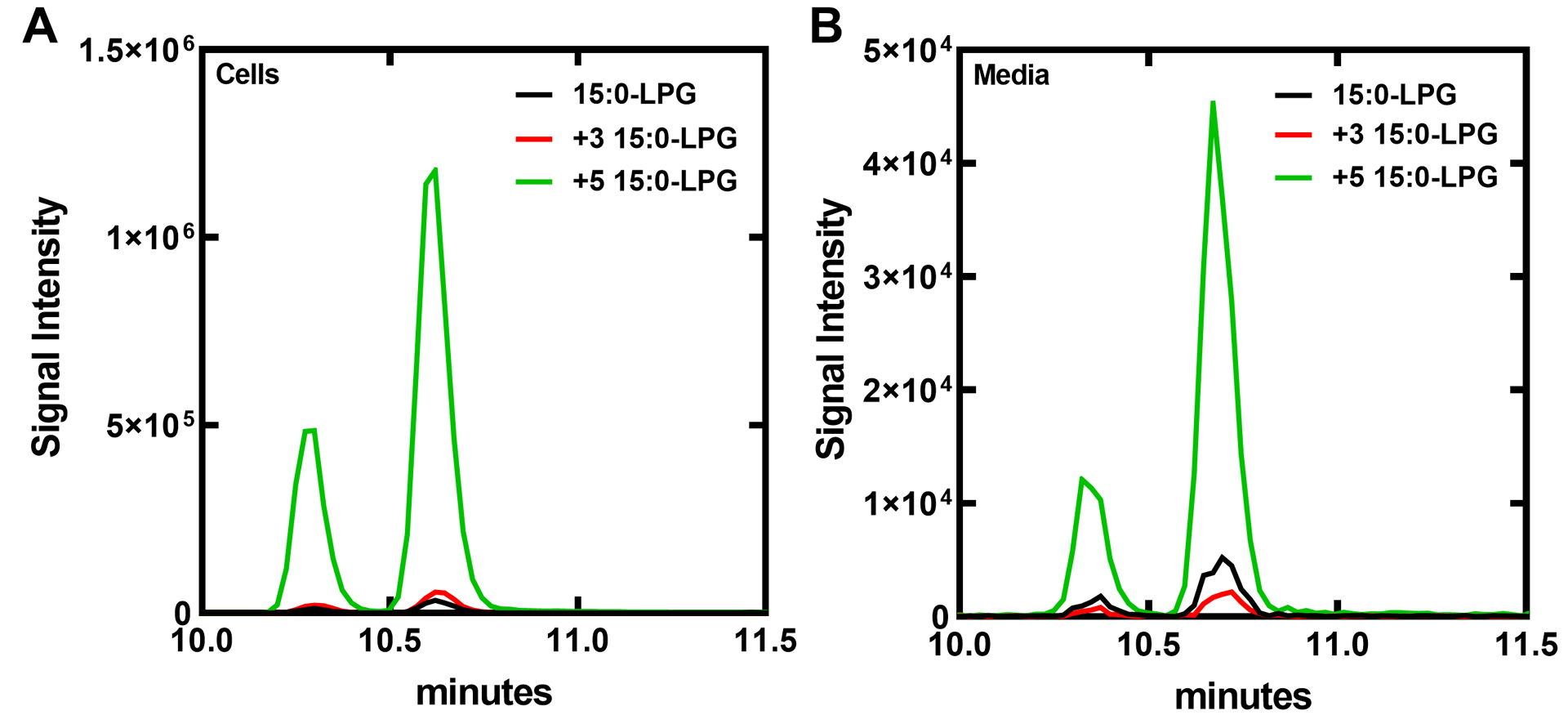

Supplement: FIG S2 [file msphere.00031-23-s0004.tif]

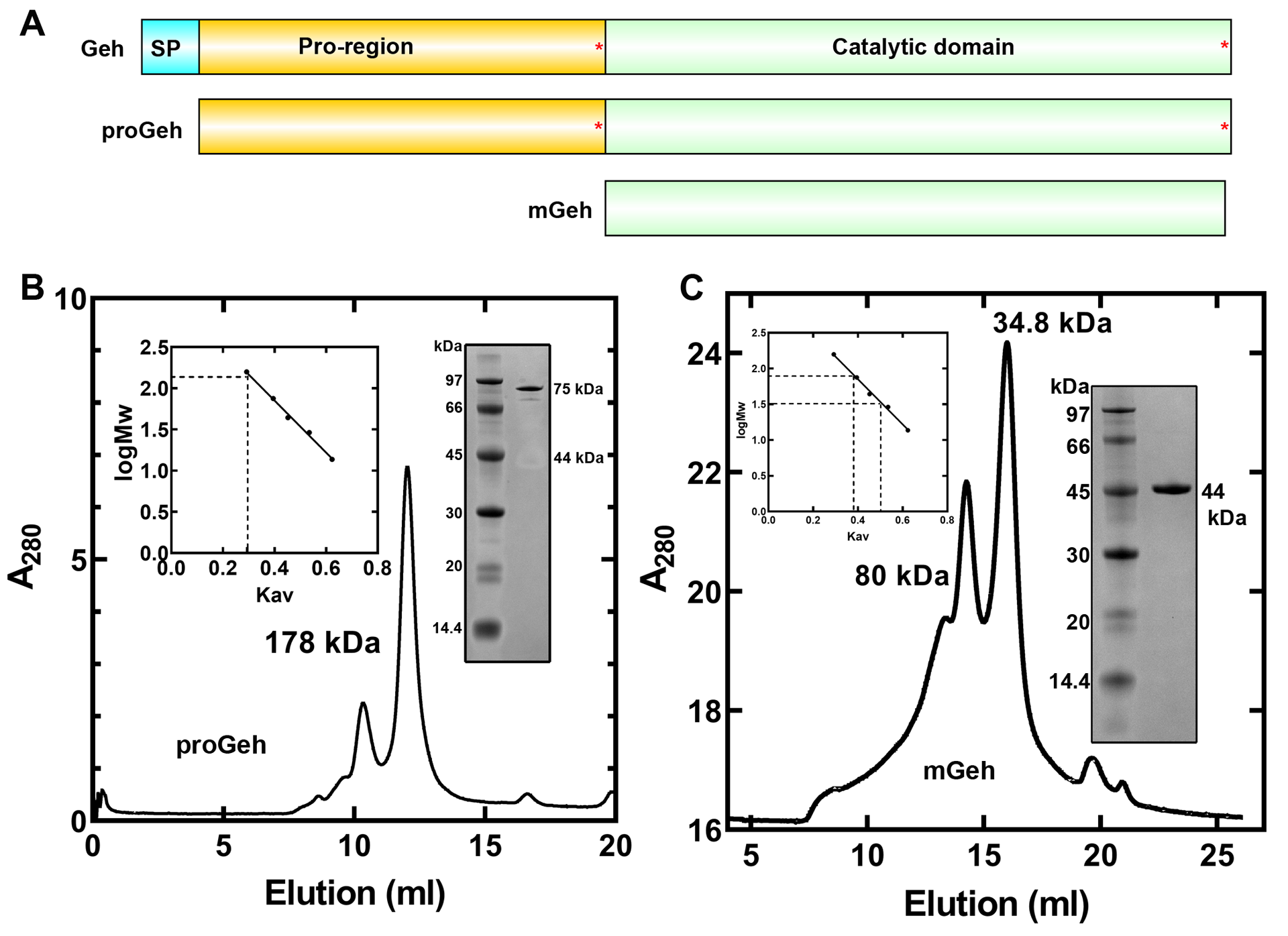

Supplement: FIG S3 [file msphere.00031-23-s0005.tif]

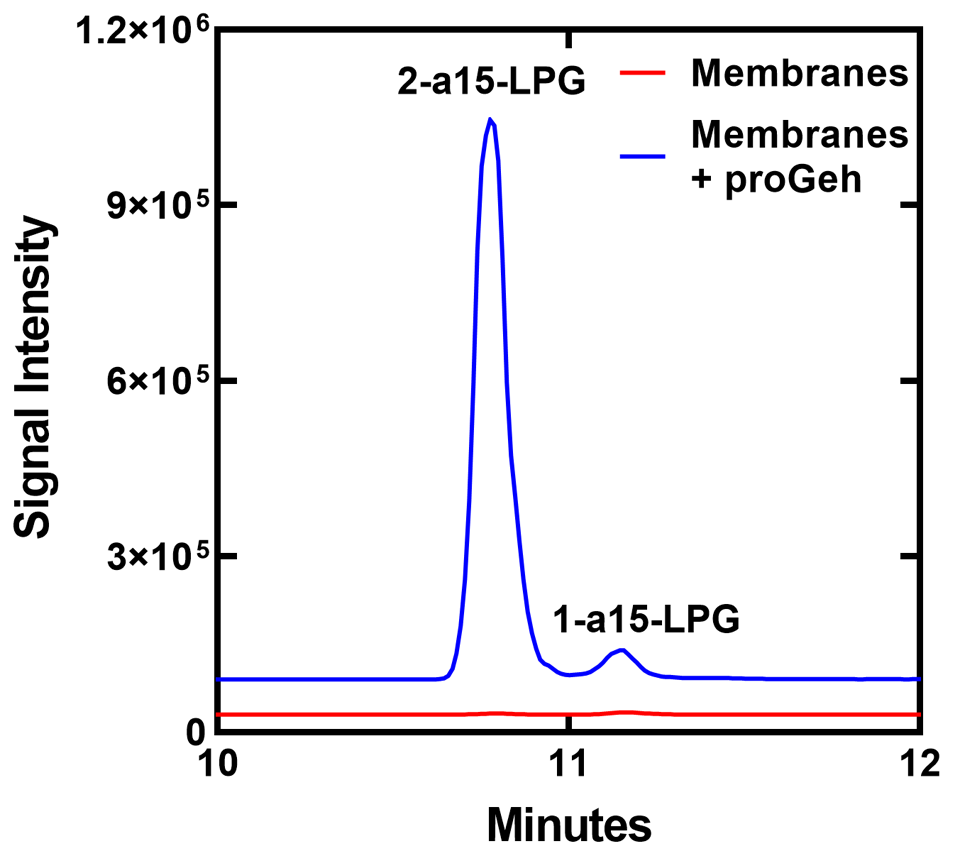

Supplement: FIG S4 [file msphere.00031-23-s0006.tif]

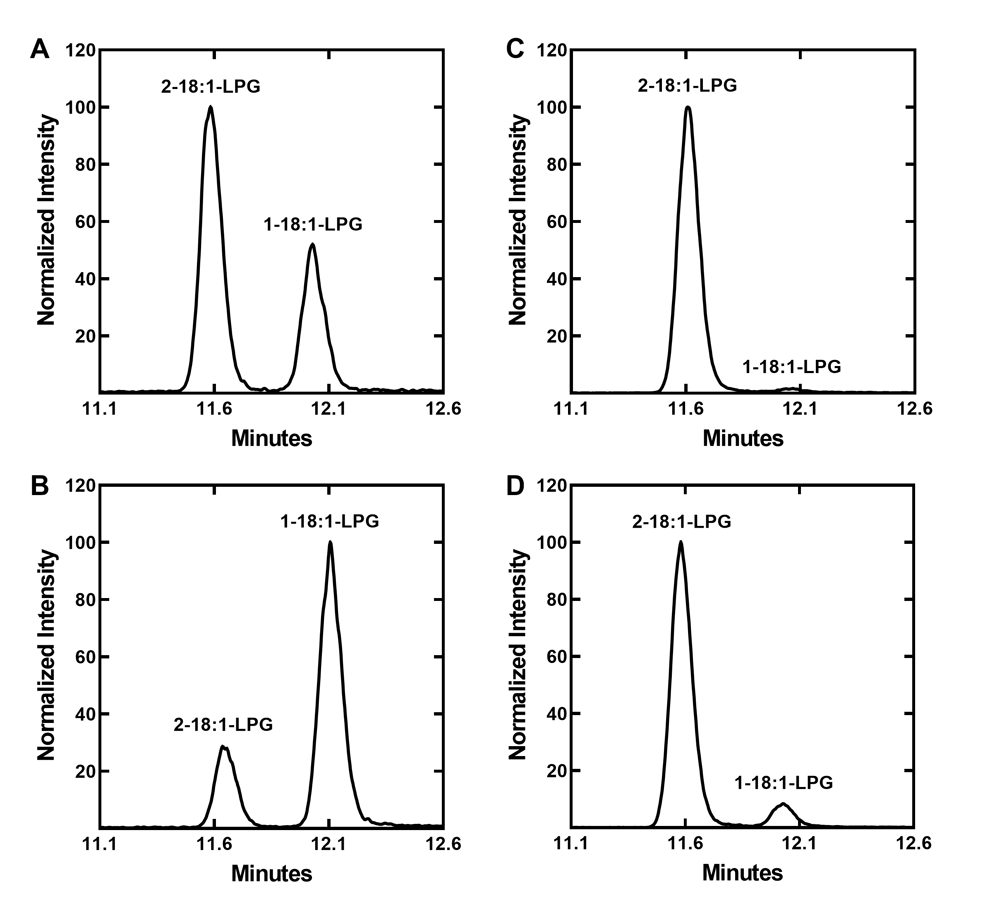

Supplement: FIG S5 [file msphere.00031-23-s0007.tif]

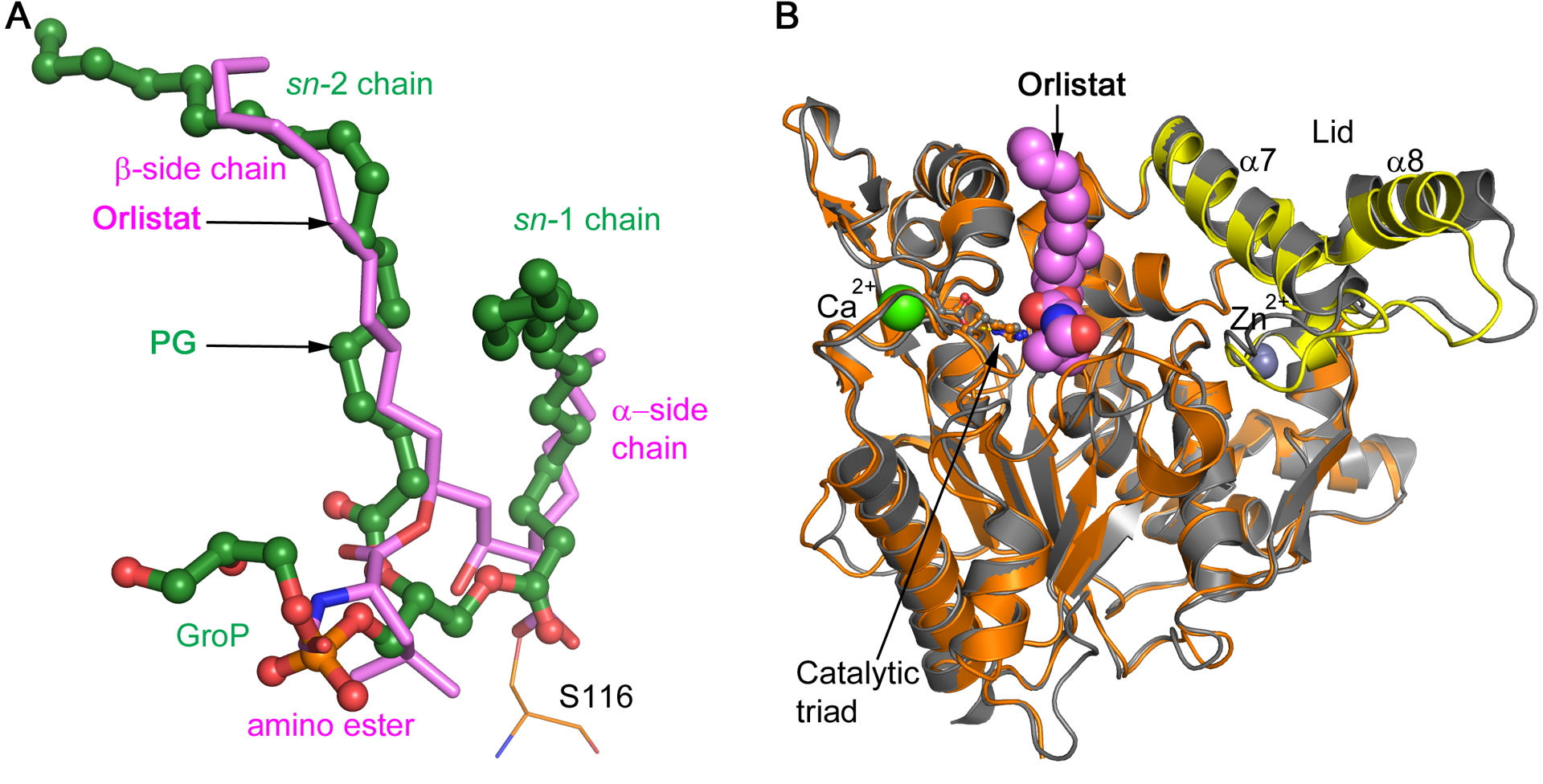

Supplement: FIG S6 [file msphere.00031-23-s0008.tif]
