# Supplementary material for: The Phospholipase A1 Activity of Glycerol Ester Hydrolase (Geh) Is Responsible for Extracellular 2-12(S)-Methyltetradecanoyl-Lysophosphatidylglycerol Production in Staphylococcus aureus
Source: mSphere. 2023 Mar 28;8(2):e00031-23. doi: 10.1128/msphere.00031-23 (PMC10117073; doi:10.1128/msphere.00031-23)
Supplement: TABLE S1 [file msphere.00031-23-s0001.docx]

**Table S1**. Strains and plasmids used in this work.

| **Strain** | **Genotype** | **Description** | **Source** |
| --- | --- | --- | --- |
| AH1263 | USA300 ErmS (LAC) | USA300 CA-MRSA strain LAC | (1) |
| PDJ171 | Δ*geh* | *geh* gene deletion in AH1263 | (2) |
| JE2 | JE2 USA300 | USA300 CA-MRSA strain LAC | (3) |
| NE1360 | *mprF*::φNΣ | *mprF* knockout | (3) |
| NE338 | *SAUSA300_2603*::φNΣ | Lip inactivation | (3) |
| NE1775 | *SAUSA300_0320*::φNΣ | Geh inactivation | (3) |
| NE104 | *SAUSA300_0641*::φNΣ | SAL3 inactivation | (3) |
| **Plasmids** |  | **Description** | **Source** |
| pPJ628 |  | pET28a proGeh | Present study |
| pPJ650 |  | pET28a mGeh | Present study |
| pPJ480 |  | pCM28 with SarA P1 promoter | (2) |
| pPJ630 |  | pPJ480 expressing Geh | (2) |

1. Kreiswirth BN, Lofdahl S, Betley MJ, O'Reilly M, Schlievert PM, Bergdoll MS, Novick RP. 1983. The toxic shock syndrome exotoxin structural gene is not detectably transmitted by a prophage. Nature 305:709-712. <https://doi.org/10.1038/305709a0>

2. Radka CD, Batte JL, Frank MW, Rosch JW, Rock CO. 2021. Oleate hydratase (OhyA) is a virulence determinant in *Staphylococcus aureus*. Microbiol Spectr 9:e0154621. <https://doi.org/10.1128/Spectrum.01546-21>

3. Bose JL, Fey PD, Bayles KW. 2013. Genetic tools to enhance the study of gene function and regulation in *Staphylococcus aureus*. Appl Environ Microbiol 79:2218-2224. <https://doi.org/AEM.00136-13>
