# Supplementary material for: The Phospholipase A1 Activity of Glycerol Ester Hydrolase (Geh) Is Responsible for Extracellular 2-12(S)-Methyltetradecanoyl-Lysophosphatidylglycerol Production in Staphylococcus aureus
Source: mSphere. 2023 Mar 28;8(2):e00031-23. doi: 10.1128/msphere.00031-23 (PMC10117073; doi:10.1128/msphere.00031-23)
Supplement: TABLE S2 [file msphere.00031-23-s0002.docx]

**Table S2** Mass transitions used to detect LPG molecular species by LC-MS/MS.

| LPG | Q1^a^ | Q3^b^ |
| --- | --- | --- |
| 13:0-LPG | 441.2 | 213.2 |
| 14:0-LPG | 455.2 | 227.2 |
| 15:0-LPG | 469.2 | 241.2 |
| 16:0-LPG | 483.2 | 255.2 |
| 17:0-LPG | 497.2 | 269.2 |
| 18:0-LPG | 511.2 | 283.2 |
| 18:1-LPG | 509.2 | 281.1 |
| 18:2-LPG | 507.2 | 279.1 |
| 19:0-LPG | 525.2 | 297.2 |
| 20:0-LPG | 539.2 | 311.2 |
| 20:1-LPG | 537.2 | 309.2 |
| 21:0-LPG | 553.2 | 325.2 |
| +5-13:0-LPG | 446.2 | 218.2 |
| +5-15:0-LPG | 474.2 | 246.2 |
| +5-17:0-LPG | 502.2 | 274.2 |
| +5-19:0-LPG | 530.2 | 302.2 |
| +3-13:0-LPG | 444.2 | 216.2 |
| +3-15:0-LPG | 472.2 | 244.2 |
| +3-17:0-LPG | 500.2 | 272.2 |
| +3-19:0-LPG | 528.2 | 300.2 |
| +4-14:0-LPG | 459.2 | 231.2 |
| +4-16:0-LPG | 487.2 | 259.2 |
| +4-18:0-LPG | 515.2 | 287.2 |
| +4-20:0-LPG | 543.2 | 315.2 |
| D5-17:0-LPG | 502.2 | 269.2 |

^a^ Q1 is the parent *m/z*

b Q3 is the product *m/z*
